# Two-year field data on neonicotinoid concentrations in guttation drops of seed treated maize (*Zea mays*)

**DOI:** 10.1016/j.dib.2018.10.006

**Published:** 2018-10-04

**Authors:** Detlef Schenke, Ina Patrizia Wirtz, Stefan Lorenz, Jens Pistorius, Udo Heimbach

**Affiliations:** a Julius Kühn-Institut, Institute for Ecological Chemistry, Plant Analysis and Stored Product Protection, Königin-Luise-Straße 19, 14195 Berlin, Germany; b Julius Kühn-Institut, Institute for Bee Protection, Messeweg 11/12, 38104 Braunschweig, Germany; c Julius Kühn-Institut, Institute for Plant Protection in Field Crops and Grassland, Messeweg 11/12, 38104 Braunschweig, Germany

## Abstract

We present neonicotinoid concentrations in guttation drops of commonly used maize (*Zea mays*) cultivars, germinated from seeds coated with active substances (a.s.): i) imidacloprid (IMD), ii) clothianidin (CTN) and iii) thiamethoxam (THM) over two growing seasons. In one variant clothianidin was applied as seed granule. The trial took place at the experimental fields of the Julius Kühn-Institut in Berlin in 2010 and 2011. Data from 2010 are related to a presentation of “Pesticides in guttation droplets following seed treatment – field studies” (Schenke et al., 2011) [1] presented at the SETAC North America conference and only some figures were used in the “Scientific opinion on the science behind the development of a risk assessement of plant protection products on bees (*Apis mellifera*, *Bombus* spp. and solitary bees)” (EFSA, 2012) [2]. Only parts of the data from 2011 was presented in relation to the “Exposure of Coccinellidae to guttation droplets on maize seedlings with seed or granule treatment of neonicotinoids” (Schenke and Heimbach, 2014) [3].

The article describes the study sites, the variants of treated maize seeds, sample collection and the analytical methods used to quantify the neonicotinoids and relevant metabolites of IMD (5-OH-IMD and IMD-olefine) and of THM (CTN) in guttation drop samples. The complete field data set is publicly available at the OpenAgrar repository under https://doi.org/10.5073/20180907-142020 (Schenke et al., 2018) [4].

**Specifications table**

**Table:** 

Subject area	*Environmental science*
More specific subject area	*Terrestrial ecology, ecotoxicology, environmental monitoring*
Type of data	*Table, figure*
How data were acquired	*LC-MS/MS measurements of daily sampled guttation droplets*
Data format	*Filtered data (means of duplicate measurements; concentrations of metabolites were added to the parent compound concentration, calculated by molecular masses)*
Experimental factors	*2 maize cultivars, three neonicotinoids, different pesticide formulations and application rates*
Experimental features	*Field test, in two years with different weather conditions, randomized block design, immediate residue analysis*
Data source location	*Experimental field of the Julius Kühn-Institut in Berlin, Germany*
Data accessibility	*The field data set is publicly available at the OpenAgrar repository under*https://doi.org/10.5073/20180907-142020.
Research article related to guttation	*E. Lippmann: Über das Vorkommen der verschiedenen Arten der Guttation und einige physiologische und ökologische Beziehungen. Bot. Arch. 1 (1925) 362–463.*
	*V. Girolami, L. Mazzon, A. Squartini, N. Mori, M. Marzaro, A. Di Bernardo, M. Greatti, C. Giorio, A. Tapparo: Translocation of neonicotinoid insecticides from coated seeds to seedling guttation drops: a novel way of intoxication for bees. J. Econ. Entomol. 102 (2009) pp 1808–1815.*
	*A. Schmolke, B. Kearns, B. O`Neill: Plant guttation water as a potential route for pesticide exposure in honey bees: a review of recent literature. Apidologie, (2018) online first, access 26.09.2018*.

**Value of the data**

• The dataset shows for the first time the concentration of three neonicotinoids in guttation drops of maize after seed treatment over two growing seasons under realistic climatic conditions of Central Europe.
• The dataset allows the comparison of the exposure pathway of three neonicotinoids from treated seeds into the guttation drops of maize at two seed treatment levels.
• The combination of the event-driven data allows deeper insights in the driving forces of the guttation process by linking to generally available meteorological parameters.
• The dataset allows to perform statistical analysis and modeling of occurrence of guttation and residue levels as a basis for risk assessment for bees and other terrestrial non-target organisms exposed to neonicotinoids in guttation drops possibly used as water source.

## 1 Data

The data presented in this article consist of analysed concentrations of three neonicotinoids in guttation water of maize *(Zea mays)* cultivars following seed coating at two different concentration levels. Additionally, neonicotinoid concentrations in guttation water were analysed from uncoated maize plants planted in row application together with neonicotinoid loaded granules. The data were collected under realistic climate conditions at the experimental fields of the Julius Kühn-Institut in Berlin in 2010 and 2011 at different plots. The data consist of one Excel sheet providing the results of the neonicotinoid analysis, the description of the sampling details (days without monitoring, days with and without guttation, days with rain wet leaf) and data on precipitation/irrigation and temperature. The results show the sum of imidacloprid and its relevant metabolites calculated as parent compound. The same was done for thiamethoxam and its relevant metabolite clothianidin. Guttation water of the 4 replicates was pooled to obtain a minimum amount of water for analysis at days with very low guttation. Data from 2010 are related to a presentation of “Pesticides in guttation droplets following seed treatment – field studies” (Schenke et al., 2011) [1] presented at the SETAC North America conference and only some figures were used in the “Scientific opinion on the science behind the development of a risk assessement of plant protection products on bees (Apis mellifera, Bombus spp. and solitary bees)” (EFSA, 2012) [2]. Only parts of the data from 2011 was presented in relation to the “Exposure of Coccinellidae to guttation droplets on maize seedlings with seed or granule treatment of neonicotinoids” (Schenke and Heimbach, 2014) [3]. The field data set is publicly available at the OpenAgrar repository under https://doi.org/10.5073/20180907-142020 (Schenke et al., 2018) [4].

## 2 Experimental design, materials and methods

### 2.1 Study site description

The experimental fields are located in the city of Berlin, Germany at an altitude of 45 m ASL. The climate is normally temperate (mean temperature: 9.6 °C, mean precipitation: 540 mm a^-1^) and the soil consists of slightly up to medium loamy sand (Table 1). The daily weather conditions were received from the German national meteorological service (Deutscher Wetterdienst – www.dwd.de) for the measuring station Berlin-Dahlem 10381, which is a few hundred meter away from the study area. Precipitation was measured directly on the field by an automatic rain gauge with a tipping bucket (F&C GmbH). An extreme drought period occurred in June and July 2010 making it necessary to irrigate the maize fields according to common good agricultural practice.

**Table 1:** Soil characteristic of the upper soil layer (0–30 cm). C = carbon, N = nitrogen, C_org_ = organic carbon, WRC = water retention capacity, AK_e_ = cation-exchange capacity.

	Sand	Silt	Clay	pH	C/N	C_org_	WRC	AK_e_
	%	%	%			%	%	µmol_c_ g^-1^
2010	73	19	8	6.6	11	1.07	20.53	78.73
2011	73	15	12	6.4	11	1.04	19.17	73.02

**Fig. 1:**
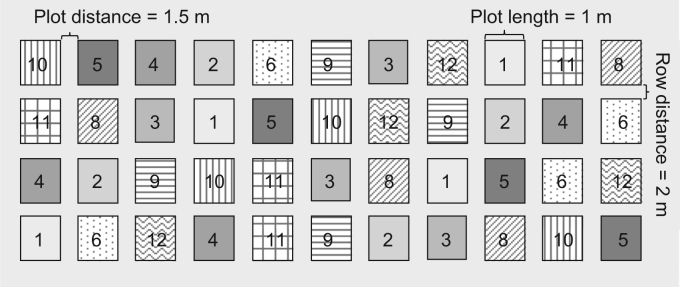
Randomized block design with four replicates per variant, each plot with a length of 1 m and a distance between plots and rows of 2 m and between plots in rows of 1.5 m. Variant 2 (2010) was replaced by variant 7 in 2011 (Table 2).

The seed treated maize cultivars and the treated granules were provided from KWS. Variants differed in active ingredient used, cultivar, application rate and pesticide formulation (Table 2). The variants were sown in a randomized design (Fig. 1) on different locations each year. No neonicotinoid pesticide was applied at the study areas at least two years before. Sowing of treated seeds and the granular application took place at May 5th 2010 and April 29th 2011 with a realistic sowing density of 100.000 seeds ha^-1^ (row space 75 cm, seed space 13 cm, depth 3–4 cm). Emergence took place before May 25th 2010 and May 9th 2011, respectively.

**Table 2:** Variants of treated maize seeds. Variant 4 (denoted in italics) refers to the treatment of row application with loaded granules. a.s. = active substance, n. d. = not determined.

Variant	cultivar	Pesticide product	Neonicotinoid		a.s. per seed	
			a.s.	Application rate, g ha^-1^	Nominal	Measured
					µg seed^-1^	
1	Amadeo	Maxim XL^a^	None	–	–	n. d.
2	Amadeo	Maxim XL^b^	None	–	–	n. d.
3	Ronaldinio	Poncho +Maxim XL^a^	Clothianidin	50	500	545
4	Ronaldinio	Santana +Maxim XL^a^	Clothianidin	50	700^c^	666^c^
5	Amadeo	Poncho +Maxim XL^a^	Clothianidin	50	500	514
6	Amadeo	Poncho +Maxim XL^b^	Clothianidin	25	250	245
7	Ronaldinio	Maxim XL^a^	None	–	–	n. d.
8	Amadeo	Cruiser +Maxim XL^a^	Thiamethoxam	50	500	547
9	Amadeo	Cruiser +Maxim XL^b^	Thiamethoxam	25	250	229
10	Amadeo	Gaucho +Maxim XL^a^	Imidacloprid	50	500	501
11	Amadeo	Gaucho +Maxim XL^b^	Imidacloprid	25	250	264
12	Amadeo	Force Zea + Maxim XL^b^	Thiamethoxam	25	350	227

a Maxim XL applied with 0.0125 l/seed unit,

b Maxim XL applied with 0.00625 l/seed unit,

c µg a. s. per 100 mg granules

### 2.2 Sample collection

Sample collection started at May 25th 2010 with the emergence of plants at BBCH growth stages 12 (two leaves unfolded [5]) and at May 9th 2011 at BBCH growth stage 10 (first leaf through coleoptile). Guttation occurred almost daily in young plants. Sampling started in the morning between 6.30 and 7.30 am once a day. The sampling period covered 88 (2010) and 103 (2011) days including 16 and 38 days with rain wet leafs and consequently no sampling, respectively, and 11 days without sample collection due to logistic constraints in both years. Sampling ended with the ripening of plants in growth stages 85 (dough stage: kernels yellowish to yellow, August 20th 2010) and in growth stage 87 (physiological maturity: black dot/layer visible at base of kernels, August 19th 2011). Guttation drops were collected randomly from different plants in the center of each plot at different heights. The guttation drops were taken from the edges of leaves (0.2–0.5 ml per sample) using a new Pasteur pipette for each replicate. Dew on the leaf surfaces with its little drops was well distinguishable from the larger guttation droplets at the edge of leaves. Samples were analysed daily after sampling from Monday to Friday. Samples collected at the weekend were frozen at −20 °C for the maximum of three days before analysis.

### 2.3 Sample analysis

The collected guttation water (10–50 µl) was added with the same volume of acetonitrile that contained the internal standard imidacloprid-d4 (concentration of 100 pg ml^-1^). Liquid chromatograph (LC, Dionex UltiMate 3000) coupled to tandem mass spectrometer (MS/MS, Qtrap 5500, AB Sciex) via an electrospray interface (ESI) was applied for the quantification of neonicotinoid concentrations in guttation water. The configuration of the LC-ESI-MS/MS system and the MS parameters are presented in Table 3, Table 4.

**Table 3:** Configuration of the LC-ESI-MS/MS system.

Liquid chromatography			
Autosampler temperature	10 °C		
Injection volume	2 µL		
Columns	Phenomenex Synergi Max RP, Luna C18		
Column temperature	70 °C		
Mobile phase A	0.1% acetic acid in methanol		
Mobile phase B	0.1% acetic acid in water		
Gradient program	Time(min)	*A* (%)	*B* (%)
	0	10	90
	3	90	10
	4	90	10
	4	10	90
	6	10	90
Flow rate	500 µL/min		
Mass spectrometry			
Mode	Positive ESI / negative ESI		
Ion spray potential	5.5– 4.5 kV		
Source temperature	450 °C		
Scan type	Multiple reaction monitoring		
Dwell time	10 ms		
Quantification	Relative peak area

**Table 4:** Parameter of the tandem mass spectrometry. DP = declustering potential, EP = entrance potential, CE = collision energy, CXP = cell exit potential, MRM = multiple reaction monitoring.

MRM	Precursor	Product	DP	EP	CE	CXP
	(m/z)		(V)			
Thiamethoxam	292	211	42	10	17	16
Clothianidin	250	169	41	10	19	12
Imidacloprid	256	209	58	10	49	6
5-OH-imidacloprid	270	46	−120	−10	−59	−6
Imidacloprid-olefine	252	205	−50	−10	−18	−23
Imidacloprid-D4	260	213	58	10	49	6

The neonicotinoids were quantified with reference standards in the solvent, and quantification followed the internal standard method. The calibration curves were linear (*r*² > 0.99) in the range of 0.1–100 pg µl^-1^ (Qtrap 5500). All data are presented as average of duplicate injections of the sample extracts. The limit of detection (LOD) was 0.1 ng ml^-1^ for all analytes.

Imidacloprid-d4 (C 14283710) as internal standard and imidacloprid (C 14283700), thiamethoxam (C 17453000) and clothianidin (C 11691700) were purchased from Dr. Ehrenstorfer GmbH, Germany. The metabolites imidacloprid-olefine and 5-hydroxy-imidacloprid were derived from Bayer CropScience, Germany. Aceton, acetonitrile and methanol were used as pesticide grade specification. Deionized water for LC was produced using an Arium 611 UV system (Sartorius, Germany).

### 2.4 Validation of seed coating rate

The neonicotinoid rate applied to the seeds was checked before the field tests. Ten seeds of each seed cultivar were separately sonicated in aceton (Sonorex Super 10P - Bandelin) in a 50 ml closed glass bottle for 10 min. An aliquot of the solution was reduced to dryness after 1 hour, redissolved together with the internal standard imidacloprid-d4 in acetonitrile and filtered (PTFE, 0.2 µm, Roth). LC-ESI-MS/MS (PE Sciex API 2000) was used for quantification. The calibration curves were linear (*r*² > 0.99) in the range of 1–1000 pg µl^-1^. All seeds were coated with concentrations in the range of the nominal concentrations (see Table 2). No neonicotinoids and metabolites (LOD < 0.1 µg seed^-1^) were detected in untreated seeds. The additionally coated fungicides were not analysed.

### 2.5 Data outline

**Fig. 2:**
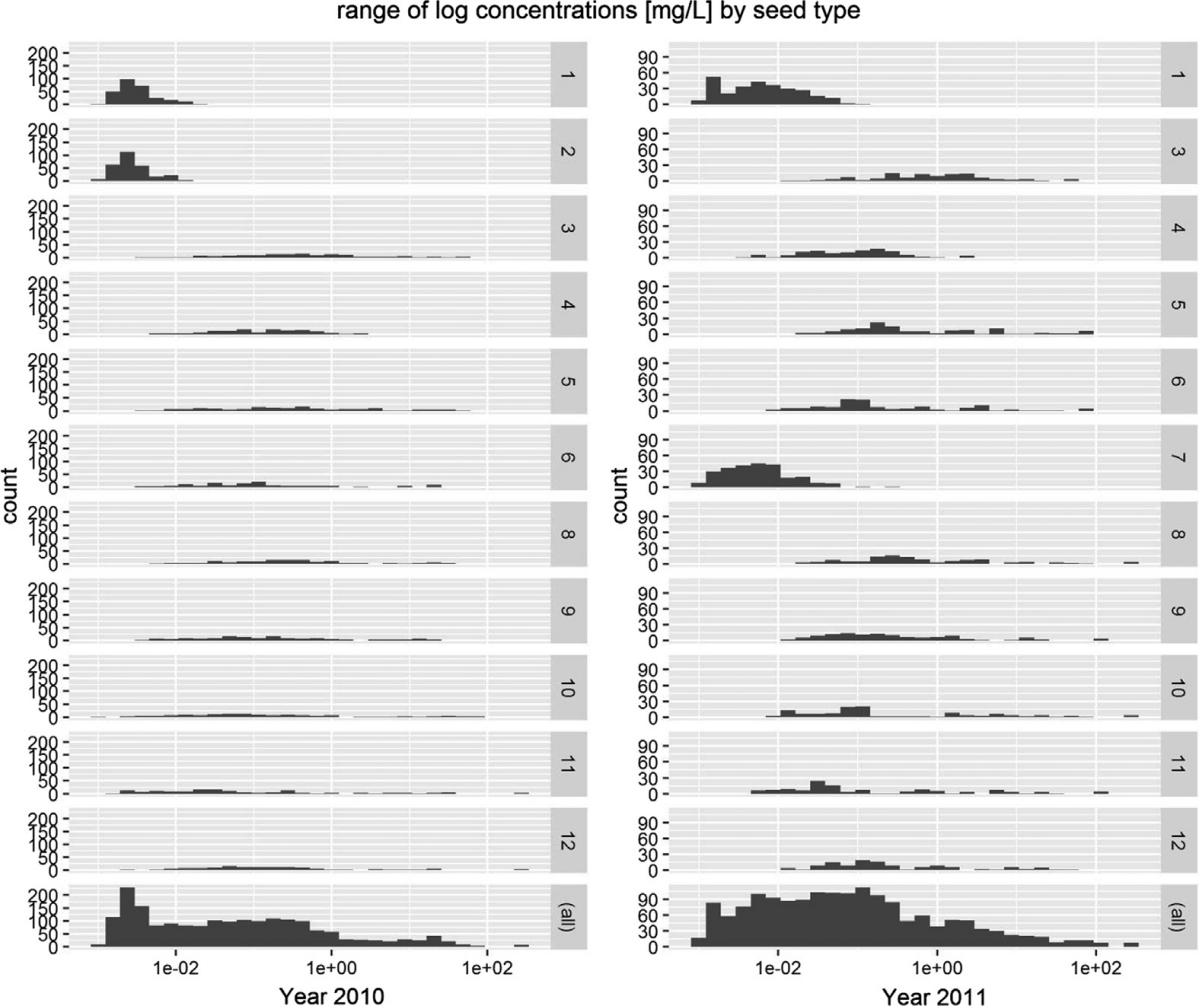
Number of observations of active substance detection and concentration range [mg/L] of all active substances measured in 2010 (left panel) and 2011 (right panel). Right hand numbers denote variants of treated maize seeds from Table 2 (control variants 1 and 2 (2010) as well as 1 and 7 (2011)). Please note differing scales between 2010 and 2011.

The total data set available under https://doi.org/10.5073/20180907-142020 consists of 10,140 observations (individual observation of each replicate in all variants at all days except for the days when no monitoring was conducted) (Table 5). In total, 4122 guttation events (number of all replicates in all variants with sampled guttation drops) were observed which corresponds to 36% of the data set (Table 5). Concentrations of active substances were above the LOD in 86% of cases in which guttation occurred including control treatments. The range of concentrations > LOD measured in all 12 variants is shown in Fig. 2.

**Table 5:** Number of observations with percentages in brackets. CTN = clothianidin, THM = thiamethoxam, IMD = imidacloprid, a.s. = active substance.

**Year**		**Guttation = Yes**	
2010	5280 (46.1)	CTN	1630 (39.6)
2011	6180 (53.9)	THM	1377 (33.4)
**Guttation**		IMD	1115 (27.0)
Yes	4122 (36.0)	a.s. < LOD	565 (13.7)
No	2778 (24.2)	a.s. > LOD	3557 (86.3)
Rain	3240 (28.3)		
No monitoring	1320 (11.5)

We applied a tobit regression model (R package VGAM [6]; R Core Team [7]) to the data set to account for censoring in the dependent variable in the data set (resulting from non-detects < LOD) with measured concentration irrespectively of the type of neonicotinoid as dependent variable. This was done in order to receive a first rough glance of the data structure. Values < LOD were set to 0 for this purpose and only guttation events were considered. Please note that DAA (days after application) and BBCH growth stage are highly correlated (spearman rank correlation, rho = 0.99) leading to the exclusion of BBCH growth stage from the tobit model, whereas there is a less strong correlation of DAA with temperature (spearman rank correlation, rho = 0.22). The tobit model shows that DAA and temperature have significant effect on neonicotinoid concentrations in the data set, while there is no significant effect of precipitation (Table 6). There is also a significant difference in the data between years, and all variants of treated maize seeds differ significantly from the control variant 1 (Table 6). No significant differences were found between the three control variants 1, 2 and 7. The data set needs further evaluation.

**Table 6:** Results of the tobit regression model applied to the data set. DAA = days after application. For seed variants see Table 2.

	Estimate	Standard error	*Z* value	Pr (>|z |)	
Intercept 1	3.08535	1.95111	1.583	0.113803	
Intercept 2	2.94896	0.01703	173.200	< 2e−16	***
DAA	−0.13532	0.01526	−8.865	< 2e−16	***
Temperature	−0.28538	0.10057	−2.838	0.004544	**
Precipitation	0.02775	0.13399	0.207	0.835952	
2010 vs 2011	2.62003	0.73538	3.563	0.000367	***
Seed-2	−0.46058	1.35074	−0.341	0.733115	
Seed-3	8.48015	1.48073	5.727	1.02e−08	***
Seed-4	5.82434	1.54565	3.768	0.000164	***
Seed-5	10.50377	1.45531	7.218	5.29e−13	***
Seed-6	8.18476	1.46982	5.569	2.57e−08	***
Seed-7	−0.87533	1.43983	−0.608	0.543230	
Seed-8	12.16796	1.45240	8.378	< 2e−16	***
Seed-9	8.62791	1.46656	5.883	4.03–09	***
Seed-10	14.31597	1.43558	9.972	< 2e−16	***
Seed-11	12.76715	1.44615	8.828	< 2e−16	***
Seed-12	10.96962	1.45656	7.531	5.03e−14	***

Signif. codes: 0 ‘***’ 0.001 ‘**’ 0.01 ‘*’ 0.05 ‘.’ 0.1.
